# Identification and Characterization of Chalcone Isomerase Genes Involved in Flavonoid Production in *Dracaena cambodiana*

**DOI:** 10.3389/fpls.2021.616396

**Published:** 2021-02-25

**Authors:** Jiahong Zhu, Wan Zhao, Rongshuang Li, Dong Guo, Huiliang Li, Ying Wang, Wenli Mei, Shiqing Peng

**Affiliations:** ^1^Key Laboratory of Biology and Genetic Resources of Tropical Crops, Ministry of Agriculture, Institute of Tropical Bioscience and Biotechnology, Chinese Academy of Tropical Agricultural Sciences, Haikou, China; ^2^Hainan Academy of Tropical Agricultural Resource, Chinese Academy of Tropical Agricultural Sciences, Haikou, China

**Keywords:** *Dracaena cambodiana*, chalcone isomerase, flavonoid, biosynthesis, gene expression

## Abstract

Dragon’s blood is a traditional medicine in which flavonoids are the main bioactive compounds; however, the underlying formation mechanism of dragon’s blood remains largely poorly understood. Chalcone isomerase (CHI) is the key enzyme in the flavonoid biosynthesis pathway. However, *CHI* family genes are not well understood in *Dracaena cambodiana* Pierre ex Gagnep, an important source plant of dragon’s blood. In this study, 11 *CHI* family genes were identified from *D. cambodiana*, and they were classified into three types. Evolutionary and transcriptional profiling analysis revealed that *DcCHI1* and *DcCHI4* might be involved in flavonoid production. Both *DcCHI1* and *DcCHI4* displayed low expression levels in stem under normal growth conditions and were induced by methyl jasmonate (MeJA), 6-benzyl aminopurine (6-BA, synthetic cytokinin), ultraviolet-B (UV-B), and wounding. The recombinant proteins DcCHI1 and DcCHI4 were expressed in *Escherichia coli* and purified by His-Bind resin chromatography. Enzyme activity assay indicated that *DcCHI1* catalyzed the formation of naringenin from naringenin chalcone, while DcCHI4 lacked this catalytic activity. Overexpression of *DcCHI1* or *DcCHI4* enhanced the flavonoid production in *D. cambodiana* and tobacco. These findings implied that *DcCHI1* and *DcCHI4* play important roles in flavonoid production. Thus, our study will not only contribute to better understand the function and expression regulation of *CHI* family genes involved in flavonoid production in *D. cambodiana* but also lay the foundation for developing the effective inducer of dragon’s blood.

## Introduction

Flavonoids are a large and diverse group of plant secondary metabolites widely present in plants. They are known to participate in a large number of physiological and biochemical processes including photosynthesis, respiration, growth and development, and plant defense against various stresses (Przysiecka et al., 2015). Moreover, flavonoids have multiple pharmacological activities and play important roles in human health and diet (Przysiecka et al., 2015; Borovaya and Klykov, 2020). Chalcone isomerase (CHI, EC5.5.1.6.) is a key enzyme involved in the flavonoid biosynthesis, which catalyzes the conversion of chalcones to flavanones leading to the different subgroups of flavonoid compounds (Yin et al., 2019; Nabavi et al., 2020). The CHI super-family can be divided into four subfamilies depending on their phylogenetic relationships and functions (Ralston et al., 2005). Type I and type II proteins have CHI enzymatic activity and are known as the *bona fide* CHIs. Type I CHIs, ubiquitous in plants, exclusively isomerize naringenin chalcone to form (2*S*)-naringenin (Shimada et al., 2003). Type II CHIs, specific in leguminous plants, can convert both 6′-hydroxychalcone and 6′-deoxychalcone to (2*S*)-naringenin and (2*S*)-liquiritigenin (Ralston et al., 2005; Cheng et al., 2018). In addition to the *bona fide* CHIs, type III and type IV CHIs exhibiting no CHI activity are also found in plants. Type III proteins, widely distributed in land plants and green algae, are shown to be fatty acid-binding proteins and involved in fatty acid metabolism in plants (Ngaki et al., 2012). Although lacking CHI cyclization activity, type IV CHIs can act as the enhancer of flavonoid production in plants (Morita et al., 2014; Jiang et al., 2015; Ban et al., 2018; Waki et al., 2020).

Dragon’s blood, a kind of red resin mainly excreted from several *Dracaena* plants, is one of the most popular and frequently used traditional medicine (Gupta et al., 2008); its formation is considered to be a physiological response of plants to environmental stresses (Wang et al., 2011; Zhu et al., 2016). Flavonoids and their oligomers are the primary chemical clusters in dragon’s blood of *Dracaena* plants (Sun J. et al., 2019). Flavonoids from *Dracaena* plants have a wide spectrum of pharmacological activities including analgesic, anticancer, antitumor, antibacterial, antifungal, anti-inflammatory, hypoglycemic activities, and cytotoxic effects (Wang et al., 2017; Sun J. et al., 2019). Unlike the typical flavonoids in other plants, flavonoids mainly found in dragon’s blood are loureirin a, loureirin b, and flavans. According to the chemical structure and proposed biosynthesis pathway of flavonoids in *Dracaena* plants, CHI is considered to play an important role in the biosynthesis of these flavonoids (Wang et al., 2011; Zhu et al., 2020). Considering the important roles of CHI in the production of flavonoids in dragon’s blood, molecular characterization and functional identification of *CHI* family genes in *Dracaena* plants are important to understand the underlying mechanisms of flavonoid production and develop the effective inducer of dragon’s blood. *Dracaena cambodiana* is an important medicinal plant that is used for the production of dragon’s blood (Luo et al., 2011). The aim of this study is to identify key *DcCHI* genes involved in flavonoid production and investigate their expression characteristics. First, 11 *CHI* family genes were identified from *D. cambodiana*. Then, a detailed analysis of evolutionary patterns, conserved domains, gene structure, and expression profiling was carried out. Moreover, enzyme activity and gene overexpression were also investigated. Our results suggested that *DcCHI1* and *DcCHI4* were involved in flavonoid production in *D. cambodiana*, and their expression was induced by methyl jasmonate (MeJA), 6-benzyl aminopurine (6-BA), ultraviolet-B (UV-B), and wounding. This study will serve as a solid foundation for further investigations into mechanisms of flavonoid production and developing the effective inducer of dragon’s blood.

## Materials and Methods

### Plant Materials and Treatments

*Dracaena cambodiana* materials used in this study were grown in plantation at the Institute of Tropical Bioscience and Biotechnology in Haikou, Hainan Province. For inducer treatment, the stems of *D. cambodiana* were injected with 10% inducer of dragon’s blood and then collected at 0, 3, and 6 days after treatment according to previous reports (Zhu et al., 2016). For tissue-specific expression, the tissues of roots, stems, leaves, flowers, and fruits of *D. cambodiana* plants were collected. Two-month-old seedlings selected based on their uniformity were used for abiotic stress treatments. For hormone treatments, 200 mM MeJA, 6-BA, and abscisic acid (ABA) were sprayed on the leaves with a handheld mist sprayer. For wounding treatment, leaves were wounded with a surgical scalpel. For UV-B radiation, plants were exposed to artificial UV-B according to previous reports (Zhu et al., 2020). Leaves were sampled from five independent plants of each treatment at 0, 3, 12, and 24 h after abiotic stress. All samples were taken and immediately frozen in liquid nitrogen and stored at −80°C. Tobacco (*Nicotiana benthamiana*) used for *Agrobacterium*-mediated transformation was grown in a controlled growth chamber at 26°C with a 16-h/8-h (light/dark) cycle.

### Identification and Sequence Analysis of *CHI* Gene Family

*CHI* genes from *Arabidopsis* and soybean (Dastmalchi and Dhaubhadel, 2015; Przysiecka et al., 2015) were used as queries in a BLASTx to search against transcriptome and genome sequence of *D. cambodiana* (Zhu et al., 2016, 2020; Ding et al., 2018). Candidate DcCHI sequences were further validated their putative protein domain signatures based on database searches PFAM^1^ and CDD.^2^ The calculated theoretical molecular weight and isoelectric point of proteins were calculated using ExPASy.^3^ Sequence alignment and phylogenetic tree were constructed using the CHI protein sequences from different plants by MEGA 9.0. The conserved motifs in DcCHI proteins were analyzed by MEME,^4^ and gene structure analysis was conducted using Gene Structure Display Server version 2.0. The promoter sequences (2,000 bp upstream of the translation initiation site) of *DcCHI* genes were extracted from *D. cambodiana* genome and submitted to *cis*-element analysis using PlantCARE^5^ online software.

### Expression Analysis

The response of *DcCHIs* to injection of the inducer was analyzed based on transcriptome data of *D. cambodiana* in previous reports (Supplementary Table 1; Zhu et al., 2016). Tissue expression, stress response expression, and overexpression analysis of *DcCHIs* were determined by quantitative real-time PCR (qRT-PCR) analysis. Total RNA from different samples were extracted using plant RNA Isolation Kit (FOREGENE, Chengdu, China) according to the manufacturer’s instructions. cDNA was synthesized from the total RNA using a PrimeScript RT reagent kit with gDNA Eraser (Takara, Dalian, China). The qRT-PCR analysis was carried out in triplicate using the Mx3005P Real-Time PCR System (Stratagene, La Jolla, CA, United States). qRT-PCR conditions were set as follows: 5 min at 95°C for initial denaturation, followed by 40 cycles of denaturation for 10 s at 94°C, annealing for 30 s at 60°C, and extension for 30 s at 72°C. The actin genes in *D. cambodiana* (Zhu et al., 2016) and tobacco (Liu et al., 2012) were used as the internal controls to normalize qRT-PCR data. Primers used in this study were listed in Supplementary Table 2.

### Functional Analysis of DcCHI1 and DcCHI4 *in vitro*

The open reading frames of *DcCHI1* and *DcCHI4* with the introduced *Eco*RI and *Sal*I sites were ligated into expression vector pET28a via the restriction sites. The resulting constructs and empty vector pET28a were introduced into *Escherichia coli coli* BL21 (DE3). Overnight cultures *E. coli* BL21 (DE3) harboring pET28a or pET28a-*DcCHI1*/*DcCHI4* were inoculated into 250 ml of Luria Bertani (LB) liquid medium containing 50 μg/ml kanamycin and grown at 37°C with shaking to an OD600 value of 0.6–0.8 followed by induction with 0.5 mM isopropyl-beta-D-thiogalactopyranoside (IPTG) at 16°C for 6 h. His-tagged DcCHI1 and DcCHI4 proteins were purified at 4°C using a His-Bind purification kit (Novagen) following the manufacturer’s protocol and finally tested by SDS-PAGE. CHI enzyme assay was performed in 200 μl total volume containing 175 μl of Tris–HCl buffer (100 mM, pH 7.6), 20 μl of purified recombinant DcCHI1or DcCHI4 protein (0.5 μg/μl), and 5 μl of naringenin chalcone (5 mM) as substrate. After incubation at 30°C for 2 min, the reactions were terminated by addition of 200 μl of ethyl acetate and extracted twice, and then centrifuged at 10,000 rpm for 10 min. The supernatant was subsequently detected by high-performance liquid chromatography (HPLC) analysis using Ultimate 3000 UHPLC system (Dionex, Thermo Fisher Scientific, Germany) as described previously (Sun W. et al., 2019).

### Transient Overexpression in *D. cambodiana*

The full-length cDNAs of *DcCHI1* and *DcCHI4* were amplified and inserted into the overexpression vector pNC-CAM1304 under the control of cauliflower mosaic virus 35S (CaMV 35S) promoter by Nimble Cloning method (Yan et al., 2020). The resulting constructs (pNC-CAM1304-*DcCHI1* and pNC-CAM1304-*DcCHI4*) and empty vector were introduced into the *Agrobacterium tumefaciens* strain GV1301 and then infiltrated into the leaves of *D. cambodiana* with a 1-ml needle syringe. Three days after infiltration, the leaves near the infiltration point were freshly collected, immediately frozen in liquid nitrogen, and used for qRT-PCR and total flavonoid content analysis.

### Overexpression in *N. benthamiana*

Leaf disks of *N. benthamiana* were transformed by *A. tumefaciens* strain GV1301 harboring the overexpression constructs and empty vector as previously described (Rajput et al., 2017). The independent transformed lines were obtained after selection with hygromycin and then confirmed by genomic DNA PCR using transgene-specific primers (Supplementary Table 2). Seeds of transgenic lines with the higher expression level of *DcCHI1* or *DcCHI4* were harvested and used for further studies. The leaves of 3-month-old plants of wild-type (WT) and transgenic tobacco lines were collected for qRT-PCR and total flavonoid content analysis.

### Analysis of Total Flavonoid Content

Total flavonoids in *D. cambodiana* and tobacco were determined using the methods described in previous reports (Lia et al., 2006). Leaf tissues of *D. cambodiana* and tobacco were ground in liquid nitrogen and digested in 80% methanol for 24 h. The extracts were clarified by centrifugation (13,000 × *g*, 30 min). The recovered supernatants were added with 10% AlCl_3_ to a final concentration of 1%, and then were measured absorbance values at 420 nm for samples. The absorbance values at 420 nm were normalized by the fresh weight of samples.

### Statistical Analysis

All the experiments were done in triplicate and repeated independently three times. The results were presented as the mean ± standard deviation (SD). The statistical significance (^∗^*P* < 0.05 and ^∗∗^*P* < 0.01, respectively) was determined using Student’s *t*-test.

## Results

### Identification and Phylogenetic Analysis of *CHI* Genes in *D. cambodiana*

Eleven different *CHI* genes were identified based on transcriptome and genome resources of *D. cambodiana*, and their sequences were submitted to GeneBank at NCBI (Table 1). The lengths of the open reading frames (ORFs) of 11 *D. cambodiana CHI* genes ranged from 642 to 1,284 bp, encoding 213–427 amino acid residues. The predicted molecular weights of the deduced CHI proteins ranged from 23.81 to 47.69 kDa, with theoretical isoelectric point (pI) ranging from 4.83 to 9.20.

**TABLE 1:** Detailed information on putative chalcone isomerase genes in *D. cambodiana*.

Gene	GenBank accession no.	ORF (bp)	Predicted protein			pI
	
			Size (aa)	Type	MW (kDa)	
*DcCHI1*	MT937330	660	219	Type I	23.86	6.44
*DcCHI2*	MT937331	720	239	Type I	24.98	4.83
*DcCHI3*	MT937332	690	229	Type I	24.16	4.84
*DcCHI4*	MT937333	642	213	Type IV	23.81	4.97
*DcCHI5*	MT937334	669	222	Type IV	24.85	6.00
*DcFAP1*	MT937335	828	275	Type III	30.82	5.10
*DcFAP2*	MT937336	822	273	Type III	30.12	9.50
*DcFAP3*	MT937337	1284	427	Type III	47.69	7.15
*DcFAP4*	MT937338	798	265	Type III	28.97	9.20
*DcFAP5*	MT937339	681	226	Type III	25.29	5.38
*DcFAP6*	MT937340	783	260	Type III	29.07	4.99

**FIGURE 1:**
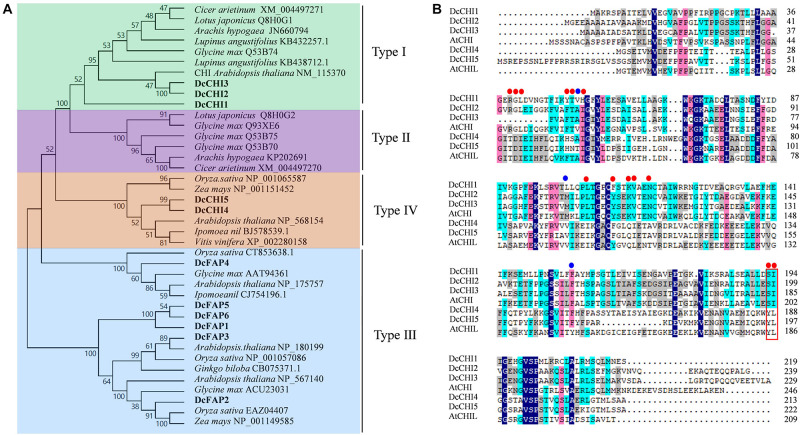
Molecular characteristics of CHI proteins. **(A)** Phylogenetic tree of CHI proteins from different plant species. The tree was constructed by neighbor-joining method with 1000 bootstrap replications using MEGA9.0. **(B)** Sequence alignment of *D. cambodiana* type I and type IV CHIs with others from *Arabidopsis thaliana*. Red and blue dots represent the active site and critical active site residues, respectively. The red box indicates residues proposed to affect substrate preference.

To investigate the phylogenetic relationship between CHI proteins in *D. cambodiana* and known CHIs from other species, a phylogenetic tree was established based on the basis of the full amino acids of CHI family proteins from different plants. The result showed that CHI family proteins from different plants belonged to four distinct groups, similar to previous reports (Ngaki et al., 2012). Eleven CHIs identified from *D. cambodiana* were classified into type I (DcCHI1, 2, and 3), type III (DcFAP1–6), and type IV (DcCHI4–5); no CHI in type II clade was found (Figure 1A). Two DcCHI homologs (DcCHI1and DcCHI2) in type I contain all the active site and critical active site residues, while DcCHI3 lacks at least 10 amino acids including three active sites (Figure 1B). As in other type IV CHI proteins, such as CHIL in *Arabidopsis* (Morita et al., 2014) and EFP in *Ipomoea nil* (Jiang et al., 2015), DcCHI4 and DcCHI5 lack almost all of the critical sites for CHI enzymatic activity (Figure 1B).

### Conserved Motifs and Intron/Exon Structure Analyses of *DcCHI* Genes

**FIGURE 2:**
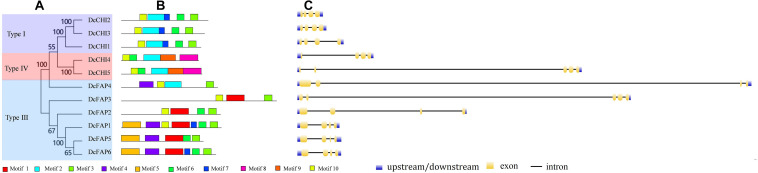
Conserved motifs and exon–intron structure analyses of CHI proteins in *D. cambodiana.* **(A)** Phylogenetic tree of DcCHIs. The tree was constructed by neighbor-joining method with 1,000 bootstrap replications using MEGA9.0. **(B)** Conserved motifs analysis of DcCHIs. Ten motifs were predicted by the online MEME program. **(C)** The exon/intron organization of *DcCHI* genes. The exons and introns are represented by yellow boxes and black lines, respectively.

To better understand classification and evolutionary patterns of DcCHIs, conserved motifs and intron/exon structure of DcCHIs were analyzed based on their evolutionary relationships. In general, most closely related members in same type exhibit common motif compositions and gene structure patterns, which may imply functional similarity between the DcCHI proteins in the same subfamily (Figure 2). For example, type I CHI subfamily proteins possessed motifs 2, 3, 6, 7, and 10, while type IV members contained motifs 2, 3, 8, 9, and 10. However, there are also exceptions among these genes. For example, each member of type III subfamily has different motif types or orders. In addition, most type III genes (except DcPAP3) had four exons, but exhibited great diversity in exon and intron length. These features suggested that functional differentiation may exist among type III subfamily members.

### Analysis of *Cis*-Elements in the *DcCHI* Promoters

**FIGURE 3:**
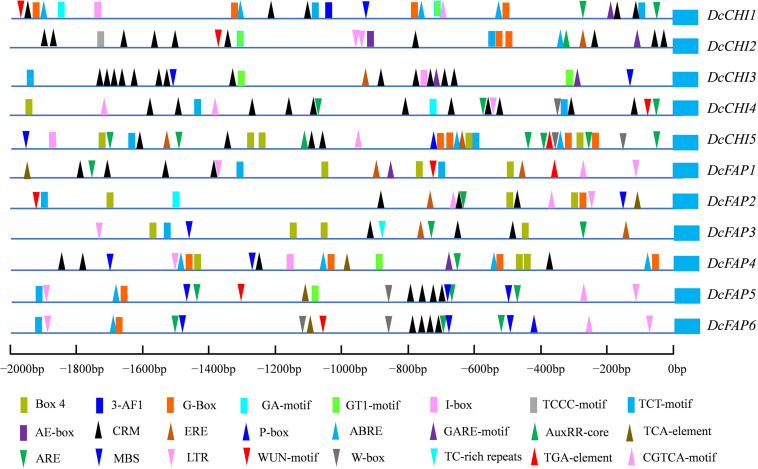
Predicted *cis*-regulatory elements of *DcCHIs*. The promoter sequences (2,000 bp upstream of the translation initiation site) of 11 *DcCHI* genes were analyzed by online analysis software PlantCARE. The blue box on the right indicates the translation initiation ATG.

To predict regulatory characteristics of *DcCHI* genes, the 2000-bp DNA fragments upstream of the ATG start codon were analyzed using PlantCARE. According to the results, the promoters of all *DcCHI* genes contained multiple light-responsive elements such as Box-4, G-Box, I-Box, 3-AF1, AE-box, GT1-motif, TCCC-motif, TCT-motif, and GA-motif (Figure 3). In addition, the promoters of all *DcCHI* genes contained at least one hormone responsive element, such as the ABRE (responsive to abscisic acid stress), CRM (responsive to cytokinin), P-box, and GARE (responsive to gibberellin) (Figure 3). Many *cis*-elements responsive to biotic and abiotic stresses such as WUN motif (wound), MBS (drought inducibility element), TC-rich repeats (defense and stress responsive element), W-box (elicitors responsive element), and LTR (low-temperature responsive element) were also found in the *DcCHI* promoters (Figure 3). Together, the presence of those *cis-*elements suggested that *DcCHIs* may be regulated by light, hormone, and abiotic stresses.

### Screening *CHI* Genes Related to Flavonoid Production in *D. cambodiana*

**FIGURE 4:**
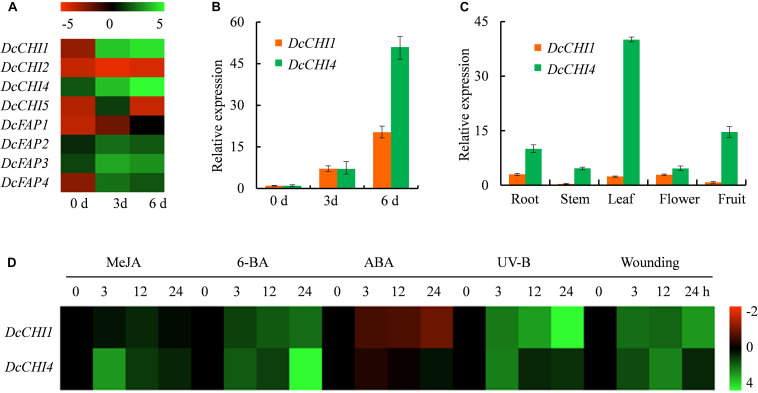
Expression profiles of *DcCHIs*. **(A)** Expression profiles of *DcCHIs* in stems of *D. cambodiana* after inducer treatment. Log2-based Fragments Per Kilobase per Million (FPKM) value for each gene was used for building the heat map. **(B)** Expression of *DcCHI1* and *DcCHI4* in stems after inducer treatment were measured by qRT-PCR. **(C)** Expression levels of *DcCHI1* and *DcCHI4* in root, stem, leaf, flower, and fruit. **(D)** Expression profiles of *DcCHI1* and *DcCHI4* in leaves under MeJA, 6-BA, ABA, UV-B, and wounding treatment were measured by qRT-PCR. Log2-based expression value for each gene was used for building the heat map.

The injection of the inducer could increase flavonoid production and the dragon’s blood formation in *D. cambodiana* (Zhu et al., 2016). To screen *CHI* genes involved in flavonoid accumulation, the expression characteristics of *DcCHIs* in stems after inducer treatment were analyzed based on our previously published transcriptome data (Zhu et al., 2016). The results revealed that 8 out of 11 *CHI* genes were expressed in stems before and after treatment, among them, five genes (*DcCHI1*, *DcCHI4*, *DcFAP2*, *DcFAP3*, and *DcFAP4*) were significantly upregulated after inducer treatment (Figure 4A). Among those upregulated *CHI* genes, *DcCHI1* and *DcCHI4* belong to type I and IV, respectively; two type genes have been shown to be involved in the accumulation of flavonoids in plants (Ngaki et al., 2012). The transcripts of *DcCHI1* and *DcCHI4* were further confirmed by qRT-PCR analysis (Figure 4B). The results suggest that *DcCHI1* and *DcCHI4* may play important roles in flavonoid production.

### Expression Analysis of *DcCHI1* and *DcCHI4*

The expression levels of *DcCHI1* and *DcCHI4* in diverse tissues (root, stem, flower, leaf, and fruit) were detected by qRT-PCR. The results showed that *DcCHI1* and *DcCHI4* were expressed in all tested organs but at different levels (Figure 4C). The *DcCHI1* was highly and similarly expressed in root, leaf, and flower, but lowly expressed in fruit and stem. The *DcCHI4* was strongly expressed in leaf, followed by fruit and root, but weakly in stem and flower. The results suggested that both *DcCHI1* and *DcCHI4* exhibited low expression levels in stem under normal growth conditions.

Since multiple stress responsive elements are located within the promoters of *DcCHI1* and *DcCHI4*, we investigated whether those stresses (MeJA, 6-BA and ABA, UV-B, and wounding) affect the expression patterns of *DcCHI1* and *DcCHI*4. The qRT*-*PCR results showed that transcripts of both *DcCHI1* and *DcCHI4* were significantly induced by 6-BA, UV-B, and wounding treatments (Figure 4D). Under exogenous MeJA treatment, the expression level of *DcCHI1* was slightly upregulated, while the expression of *DcCHI4* increased significantly (Figure 4D). Under ABA treatment, the expression of *DcCHI1* was repressed, but *DcCHI4* showed no obvious expression difference after treatment (Figure 4D).

### Functional Characterization of Recombinant DcCHI1 and DcCHI4 *in vitro*

**FIGURE 5:**
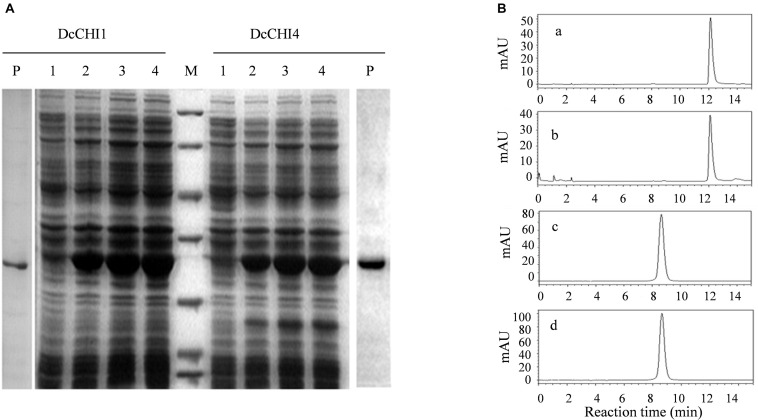
Enzymatic assays of the recombinant DcCHI1 and DcCHI4 *in vitro*. **(A)** Expression of *DcCHI1* and *DcCHI4* in *E. coli*. M, protein molecular weight marker (116.0, 66.2, 45.0, 35.0, 25.0, 18.4, 14.4 kDa); lane 1, supernatant of pET28a cell lysate; lanes 2, 3, and 4, supernatant of pET28a-DcCHI1 or -DcCHI4 cell lysates after IPTG induction for 2, 4, and 6 h, respectively; P, purified protein DcCHI1 or DcCHI4. **(B)** Enzymatic assays of the recombinant DcCHI1 and DcCHI4. **(a)** Naringenin standard; **(b–d)** HPLC elution profiles of the *in vitro* reaction products of DcCHI1(b), DcCHI4 **(c)**, and the control (empty pET28a vector) **(d)** with naringenin chalcone as the substrate.

To test whether *DcCHI1* and *DcCHI4* encode functional CHI enzyme, the coding regions of two genes were expressed in *E. coli* BL21 using pET28a expression vector. The recombinant proteins were extracted and purified by His-Bind resin chromatography. The SDS-PAGE analysis showed that the purified recombinant proteins bands were around 28 kDa, which were in agreement with the predicted values (Figure 5A). Enzyme activity assayed by HPLC indicated that recombinant DcCHI1 could catalyze the formation of naringenin with naringenin chalcone as the substrate, while recombinant DcCHI4 and control reactions did not produce naringenin using the same substrate and buffer (Figure 5B). The results indicate that DcCHI1 shows type I CHI activity catalyzing the conversion of naringenin chalcone to naringenin, while DcCHI4 lacks the CHI activity.

### Overexpression of *DcCHI1* and *DcCHI4* in *D. cambodiana* and Tobacco

**FIGURE 6:**
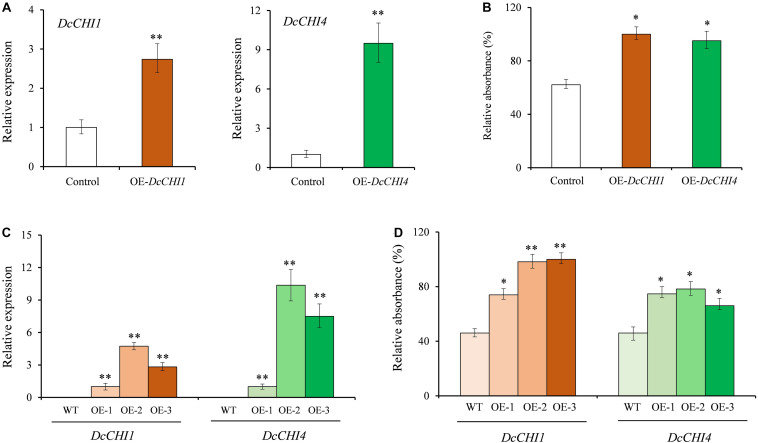
Overexpression of *DcCHI1* and *DcCHI4* in *D. cambodiana* and tobacco. **(A)** Expression levels of *DcCHI1* and *DcCHI4* in *D. cambodiana* leaves infiltrated with the overexpression constructions (pNC-CAM1304-*DcCHI1* and pNC-CAM1304-*DcCHI4*) and empty vector (Control) were measured by qRT-PCR. The expression in control is set as 1. **(B)** Flavonoid content in control and transient overexpression lines. **(C)** Expression levels of *DcCHI1* or *DcCHI4* in wild-type (WT) and overexpression tobacco lines were measured by qRT-PCR. The expression in OE-1 is set as 1. **(D)** Flavonoid content in WT and overexpression tobacco lines. Data represent the mean of three independent biological replicates. Comparisons between groups were performed by Student’s *t*-test (*P < 0.05; **P < 0.01).

Due to the lack of an efficient genetic transformation system, we performed a transient overexpression of *DcCHI1* and *DcCHI4* in *D. cambodiana* leaves to investigate their functions. After 3 days of infiltration, the relative expression levels of *DcCHI1* and *DcCHI4* increased by 1.7 and 8.4 times compared to the control, respectively (Figure 6A). Similarly, the total flavonoid content in leaves transiently overexpressing *DcCHI1* and *DcCHI4* showed significant increase by 61 and 53% as compared to the control (Figure 6B).

To verify whether *DcCHI1* and *DcCHI4* enhance flavonoid production in other plants, we overexpressed them in tobacco. Three independent transgenic lines with the higher expression level of *DcCHI1* or *DcCHI4* were chosen for flavonoid content analysis (Figure 6C). According to the results, the flavonoid production in three overexpressing *DcCHI1* and *DcCHI4* lines increased 0.6- to 1.1-fold and 0.4- to 0.7-fold than control, respectively (Figure 6D).

## Discussion

Flavonoids represent the main active chemical constituents of dragon’s blood and original plants, which have multiple pharmacological activities; however, the underlying mechanisms of flavonoid biosynthesis largely remain unclear (Zhu et al., 2016). CHI is a key enzyme involved in the biosynthetic pathway of flavonoids, which catalyzes the conversion of chalcones to flavanones (Ngaki et al., 2012). To investigate the formation mechanism of flavonoids in *Dracaena* plants, *CHI* gene family was identified and characterized in *D. cambodiana*. CHI-fold proteins are encoded by a small gene family, the number of which varies depending on the species. For example, *Arabidopsis thaliana* has only five genes encoding CHI-fold proteins (Ngaki et al., 2012), while 12 and 8 *CHI* family genes have been described in *Glycine max* (Dastmalchi and Dhaubhadel, 2015) and *Lupinus angustifolius* (Przysiecka et al., 2015). In this study, 11 *CHI* family genes were identified from *D. cambodiana*, and they were classified into three types: type I (*DcCHI1-3*), III (*DcFAP1-6*), and IV (*DcCHI4-5*). Type II *CHI* genes, mainly existing in leguminous plants, were also not found in *D. cambodiana*.

Injecting *D. cambodiana* stems with the inducer of dragon’s blood can accelerate the formation of dragon’s blood and promote the flavonoid production (Zhu et al., 2016). Our results showed that five genes (*DcCHI1*, *DcCHI4*, *DcFAP2*, *DcFAP3*, and *DcFAP4*) were significantly induced by the inducer, correlating with the accumulation of flavonoids, which suggested that these genes may participate in flavonoid production. Considering the important role of type I, II, and IV CHIs in the accumulation of flavonoids, we predicted that type I member *DcCHI1* and type IV member *DcCHI4* may play important roles in flavonoid production. Accumulation of flavonoids is usually associated with related gene expression (Petrussa et al., 2013; Liang et al., 2019). In this study, we found that both and *DcCHI1* and *DcCHI4* display low expression levels in stem under normal growth and rapidly increase after injecting the inducer. This result is consistent with the fact that flavonoids are very low in the stems of *Dracaena* plants and strongly increase after formation of dragon’s blood resin (Zhu et al., 2016). Similar expression results of other genes related to flavonoid synthesis were also obtained in previous studies (Wang et al., 2014; Chen et al., 2016; Zhu et al., 2016, 2018). Flavonoid synthesis and related function genes are often regulated by environmental stresses and plant hormones (Petrussa et al., 2013; Zoratti et al., 2014; Henry-Kirk et al., 2018). It has been proven that wounding and 6-BA can induce accumulation of flavonoids and expression of flavonoid biosynthesis-related genes in *D. cambodiana* (Yang et al., 2009; Wang et al., 2015; Zhu et al., 2016). Promoter analysis revealed that promoter regions of both *DcCHI1* and *DcCHI4* contain various *cis*-elements responsive to environmental stresses and plant hormones such as wound responsive element WUN-motif, anaerobic response element ARE, MeJA responsive element CGTCA-motif, cytokinin responsive element CRM, and multiple light responsive elements. The qRT-PCR assays further confirmed that the expression levels of *DcCHI1* and *DcCHI4* were upregulated by MeJA, 6-BA, UV-B, and wounding treatments, suggesting that those environmental stresses and plant hormones may promote flavonoid biosynthesis and induce dragon’s blood formation by upregulating flavonoid biosynthesis-related genes such as *DcCHI1* and *DcCHI4.*

Studies have found that type I CHIs are the *bona fide* CHIs with enzymatic activity converting naringenin chalcone to naringenin (Ni et al., 2020); overexpression of type I *CHI* genes increases the content of total flavonoid in various plants (Muir et al., 2001; Li et al., 2006; Park et al., 2011; Chung et al., 2019; Liu et al., 2019; Sun W. et al., 2019; Dare et al., 2020; Ni et al., 2020). In this study, three *CHI* Type I members were found in *D. cambodiana*; *DcCHI1* may be the key isoform involved in flavonoid biosynthesis. Enzyme activity assay indicated that recombinant protein DcCHI1 catalyzed cyclization of naringenin chalcone to naringenin *in vitro*. Overexpression of *DcCHI1* enhanced the flavonoid production in *D. cambodiana* and tobacco. Thus, these results indicate that DcCHI1 shows a typical type I CHI-cyclization activity and participates in the biosynthesis of flavonoids.

Type IV CHI proteins contain amino acid substitutions in several catalytic residues of *bona fide* CHI proteins and show no CHI activity (Ngaki et al., 2012). However, type IV CHI proteins have been confirmed to function as enhancers in the flavonoid pathway, such as EFP in *Ipomoea nil* (Morita et al., 2014) and CHIL in *A. thaliana* (Jiang et al., 2015). Here, two type IV *CHI* genes were identified in *D. cambodiana*, transcriptional profiling analysis revealed that *DcCHI4* may be involved in flavonoid accumulation. *DcCHI4* lacked most of the key catalytic residues, and recombinant *DcCHI4* did not metabolize naringenin chalcone to naringenin *in vitro*. On the other hand, overexpression of DcCHI4 enhanced the flavonoid production in *D. cambodiana* and tobacco. These results indicate that DcCHI4 promotes flavonoid production in spite of lacking catalytic activity, which is similar to previous reports (Ngaki et al., 2012; Morita et al., 2014; Jiang et al., 2015). Although type IV CHI proteins act as enhancers in the flavonoid pathway, the underlying detailed mechanism is still unclear (Morita et al., 2014; Jiang et al., 2015). Previous studies have shown that type IV CHI protein can interact with CHI and chalcone synthase (CHS) seems to function as an activator of these enzymes (Jiang et al., 2015; Ban et al., 2018). Recent studies indicated that type IV CHI protein acts as a rectifier rather than an activator of CHS, which enhances 2′,4,4′,6′-tetrahydroxychalcone production and decreases *p*-coumaroyl triacetic acid lactone formation, thereby rectifying substrates from the general phenylpropanoid pathway to the flavonoid biosynthesis (Waki et al., 2020). However, whether DcCHI4 promotes flavonoid production in *D. cambodiana* by similar mechanisms still needs further research.

## Conclusion

In the present study, 11 *CHI* family genes were identified from *D. cambodiana*. Evolutionary and transcriptional profiling analysis revealed that *DcCHI1* and *DcCHI4* isoforms may be involved in flavonoid production. Both *DcCHI1* and *DcCHI4* were induced by MeJA, 6-BA, UV-B, and wounding treatments. Enzyme activity assay indicated that DcCHI1 catalyzed the formation of naringenin from naringenin chalcone, while DcCHI4 had no catalytic activity. Overexpression of *DcCHI1* or *DcCHI4* enhanced the flavonoid production in *D. cambodiana* and tobacco. Together, these results indicated that DcCHI1 and DcCHI4 participate in flavonoid production in *D. cambodiana.*The findings will lay the foundation for exploring mechanisms of flavonoid production and developing the effective inducer of dragon’s blood.

## Data Availability Statement

The original contributions presented in the study are included in the article/Supplementary Material, further inquiries can be directed to the corresponding authors.

## Author Contributions

SP and WM designed the experiments. JZ, WZ, RL, DG, HL, YW, and WM performed the experiments. JZ and SP conducted the analysis and wrote the manuscript. All authors read and approved the final manuscript.

## Conflict of Interest

The authors declare that the research was conducted in the absence of any commercial or financial relationships that could be construed as a potential conflict of interest.

## Footnotes

1 http://pfam.xfam.org/search

2 http://www.ncbi.nlm.nih.gov/Structure/cdd/wrpsb.cgi

3 http://www.expasy.org/tools/protparam.html

4 http://meme-suite.org/tools/meme

5 http://bioinformatics.psb.ugent.be/webtools/plantcare/html/
